# Changes in Plant and Grain Quality of Winter Oat (*Avena sativa* L.) Varieties in Response to Silicon and Sulphur Foliar Fertilisation under Abiotic Stress Conditions

**DOI:** 10.3390/plants12040969

**Published:** 2023-02-20

**Authors:** Erika Kutasy, Gerda Diósi, Erika Buday-Bódi, Péter Tamás Nagy, Anteneh Agezew Melash, Fanni Zsuzsa Forgács, István Csaba Virág, Attila Miklós Vad, Bekir Bytyqi, Tamás Buday, József Csajbók

**Affiliations:** 1 Institute of Crop Sciences, Faculty of Agricultural and Food Sciences and Environmental Management, University of Debrecen, H-4032 Debrecen, Hungary; 2 Institute of Food Technology, Faculty of Agricultural and Food Sciences and Environmental Management, University of Debrecen, H-4032 Debrecen, Hungary; 3 Institute of Water and Environmental Management, Faculty of Agricultural and Food Sciences and Environmental Management, University of Debrecen, H-4032 Debrecen, Hungary; 4 Institutes for Agricultural Research and Educational Farm, University of Debrecen, H-4032 Debrecen, Hungary; 5 Department of Mineralogy and Geology, Institute of Earth Sciences, Faculty of Science and Technology, University of Debrecen, H-4032 Debrecen, Hungary

**Keywords:** oats, variety response, protein content, macroelement content, microelement content

## Abstract

In order to investigate the abiotic stress (drought) tolerance of oat (*Avena sativa* L.) with silicon and sulphur foliar fertilisation treatments, and monitor the effect of the treatments on the physiology, production, stress tolerance, plant, and grain quality of winter oat varieties, a field experiment was conducted in the growing season of 2020–2021. As a continuation of our article, published in another Special Issue of Plants, in this publication we evaluate the effect of silicon and sulphur treatments on the quality of winter oats. The whole grain sulphur content was significantly different between varieties. The foliar fertiliser treatments caused greater differences in both the carbon and nitrogen, and sulphur contents in the green plant samples, compared to the differences measured in the grain. Foliar treatments had a significant effect on the sulphur content of both plant samples and grains. Significant differences in the Al, Ba, Ca, Cu, Fe, K, Mn, Mo, Na, Ni, P, Pb, Sr, and Zn contents of oat grains were measured, both between treatments and between varieties. Winter oat varieties did not respond equally to the foliar fertiliser treatments in terms of either macronutrient or micronutrient content. When P, K, Ca, Mg, and S were summarised, the highest values were in the control plots. Significant differences in protein content were identified between winter oat varieties in response to the treatments, but the varieties did not respond in the same way to different foliar fertiliser treatments. Based on our results, we recommend the use of foliar fertilisation in oats in drought-prone areas.

## 1. Introduction

In this publication, we assess the impact of silicon and sulphur treatments on the quality of winter oats as a continuation of our article, in which the effects of these treatments on yield, assimilation parameters and water use efficiency were analysed [1]. The cultivated oat, is the most economically important *Avena* species, grown throughout the world for forage production and human consumption. It is considered as a low input cereal crop, being grown in a marginal environment, with a short growth period, high drought tolerance, nutritional value, maximum forage, and grain yield production [2,3]. Oat can also provide a variety of nutritional benefits, such as carbohydrate in the form of starch; micronutrients such as iron, zinc, manganese, and copper; lipids, and vitamins, like folate and pantothenic acid [4]. Hence, the current research progress and development on oat varieties, and oat end-use products, could make a fundamental contribution to combat human diseases such as gastrointestinal problems [5,6]. Oats could be very important in the diet of people suffering celiac disease (CD), although there could be differences between the varieties, and some patients cannot tolerate oats well [7]. However, although oats provide a variety of health and economic benefits, the current production volume may not satisfy the grain and nutritional demands of the world’s ever increasing population [8]. The low production volume, and grain nutritional composition, of oats could be due to divergence in varietal response, crop nutrient management practices, and drought induced stress [9,10].

The occurrence of drought, a recurring phenomenon with major impacts on both yield and grain qualitative traits, is the most widespread climatic extreme [11]. However, while several studies have reported on the effects of climate change on grain yields, the effect on grain nutrient composition of crops, such as oat and wheat, has received scant attention, although nutrition is a critical aspect of food security [12,13]. The extent of variation in grain nutritional composition of the caryopsis could become more profound if drought events and nutrient deficiencies co-exist during the growth period of crops [14,15]. This result clearly indicates the importance of an optimal nutrient supply as a foundation for the growth, development, and improvement in functional properties of oat grain. Therefore, agronomic-based interventions, such as the exogenous application of nutrients, is needed, to reduce the adverse effect of drought and satisfy the current market and nutritional demand for oat grain.

Interventions such as nutrient management could play a critical role in food and nutritional security under a changing climate. Although plant breeding programmes also substantially contribute to the food and nutrition sector, they have had limited success in developing higher yield and stress tolerant varieties [16]. As a complimentary approach to breeding strategies, the exogenous application of nutrients such as sulphur and silicon has been widely reported to enhance overall productivity of oat under drought conditions [17,18]. A number of studies have shown that the exogenous application of silicon increases the availability of several nutrients such as N, P, K, Ca, Mg, S, Fe, Zn, Mn, Cu, B, Cl, and Ni in the rhizosphere [9,19]. This could be partly due to silicon deposition, facilitating enhanced root strength, biomass, thickness, lateral root numbers, length, volume, nodulation and fixation of nutrients such as nitrogen [20]. These anatomical and morphological alterations could further allow the roots to easily penetrate the soil for effective nutrient and water uptake [21], which could potentially improve the nutritional composition of the grain.

One of the other most important nutrients, as a potential mitigation strategy, is sulphur, aimed at ameliorating the nutrient status of both the soil and plant tissue [22]. Although oat varieties have a low sulphur requirement, its application in the form of fertiliser improves the uptake and accumulation of Mn, Zn, and Cu [23]. This positive effect could be explained by the fact that the exogenous application of sulphur containing fertilisers could help the slowdown of oxidative processes in crops, leading to an improvement in element uptake, thus most cereals enhance their viability and ameliorate the grain quality [24]. Nevertheless, silicon and sulphur mediated nutrient availability and tissue nutrient status is greatly determined by the divergence in varietal response to nutrient uptake [9,21,23]. As far as plant tissue nutrient accumulation is concerned, genetic variability among oat varieties in response to the applied nutrients should be considered. With increasing numbers of agronomic-based intervention strategies to minimise the negative impact of drought and other associated extreme events in oat production, and nutritional composition, the overall aim of this study was to monitor the effects of silicon and sulphur treatments on the physiology, production, and stress tolerance of winter oat varieties [1]:

1. to investigate the effect of treatments on the plant and grain quality parameters of winter oats;
2. to examine whether significant differences are found in the mineral composition of the varieties;
3. to examine whether there is an interaction between the varieties and the treatments, i.e., whether the varieties respond in the same way to the treatments in terms of protein and element content.

Our hypothesis was that foliar fertiliser treatments could improve the quality of oats under drought stress, and that there are differences in stress response between varieties.

## 2. Results

### 2.1. Effect of Silicon and Sulphur Treatments on the Carbon, Nitrogen, and Sulphur Content of Oat Plants and Hulled Grains

The carbon, nitrogen, and sulphur contents of plant samples collected during the growing season, at the late milk growth stage of oats, and grain samples taken at harvest were analysed. Compared with the plant samples, the grains had significantly higher carbon and nitrogen contents, while the results were variable for sulphur.

**Figure 1:**
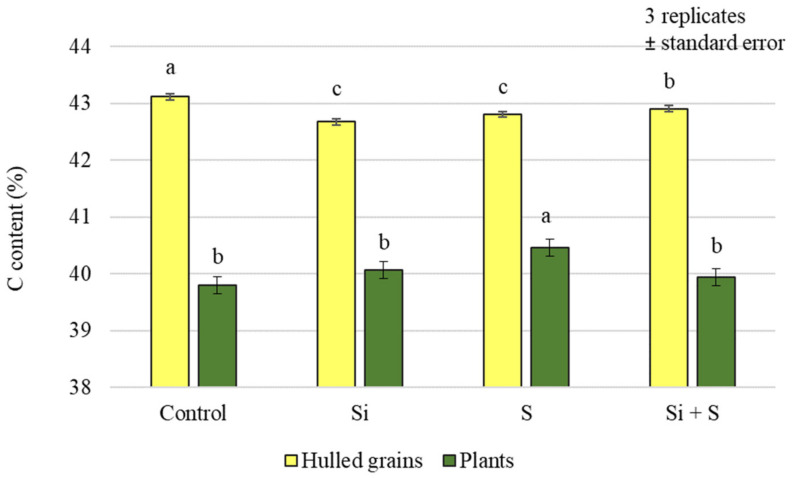
Carbon content of the winter oat plants and hulled grains as a function of the foliar fertilisation treatments; ± standard error of means. 3 replicates. The different letters show significant difference between the treatments, at *p* = 0.05 level.

**Figure 2:**
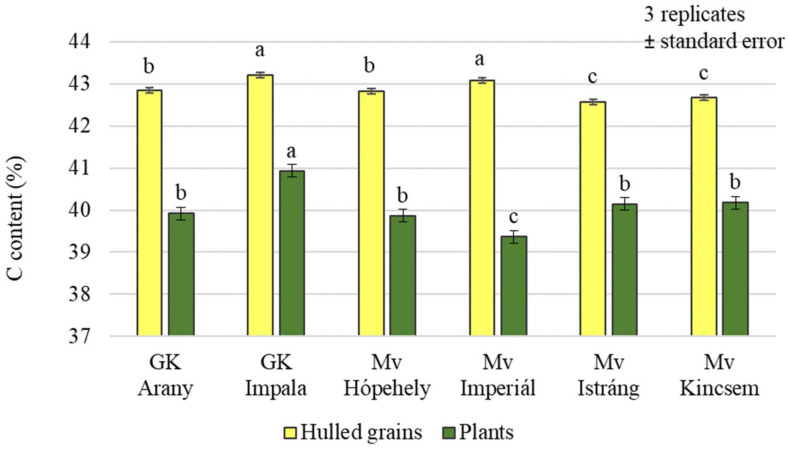
Carbon content of the winter oat varieties (plants, whole and hulled grains); ± standard error of means. 3 replicates. The different letters show significant difference between the varieties, at *p* = 0.05 level.

The analysis of the carbon content of the plants and grains showed that the effects of the treatments were not the same in the plant samples and in the grains. Only the foliar sulphur fertiliser treatment resulted in significantly higher carbon (C) levels in the plant samples. In the case of grains, a significant difference could be detected between the treatments overall, but there was no difference in the C content of grains from plots treated with the combination of Si + S foliar fertilisers (Figure 1). In the grains, the highest carbon content was measured in the control plot. The analysis of variance showed a significant difference between varieties in both plant samples and yields, with the highest carbon content in the GK Impala variety (43.21% grain; 40.93% plant) (Figure 2).

**Figure 3:**
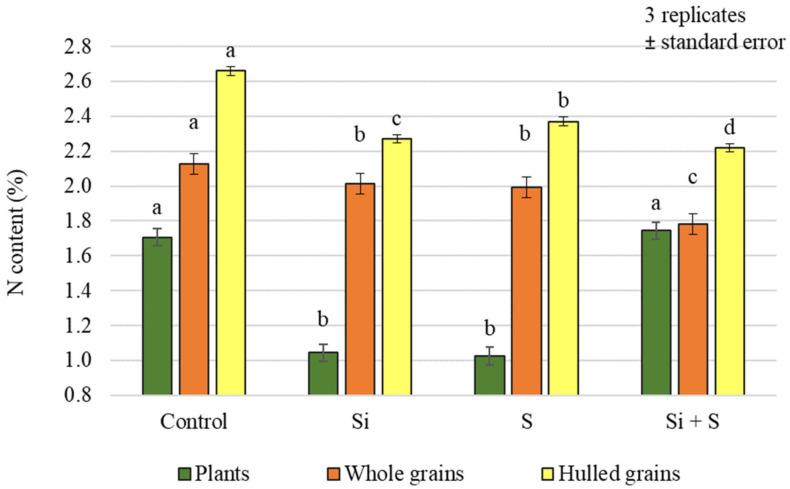
Nitrogen content of the winter oat plants, and whole and hulled grains, as a function of foliar fertilisation treatments; ± standard error of means. 3 replicates. The different letters show significant difference between the treatments, at *p* = 0.05 level.

Different foliar fertiliser treatments caused statistically significant differences in nitrogen (N) content in both plant samples and grains (Figure 3). For the plant samples, the plots receiving the Si + S treatment had the highest N content (1.743%), but this was not significantly different from the control (1.707%). For the grains, the control plots had the highest N content (whole grains: 2.124%; hulled grains: 2.659%), and the difference was significant compared to the other treatments.

**Figure 4:**
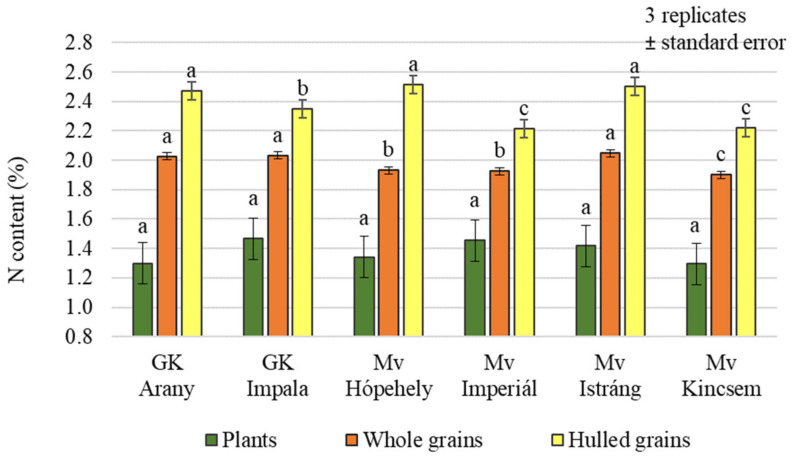
Nitrogen content of the winter oat varieties (plants, whole and hulled grains); ± standard error of means. 3 replicates. The different letters show significant difference between the varieties, separately in different plant samples, at *p* = 0.05 level.

Small differences, both in whole and hulled grains’ nitrogen contents were measured between varieties (Figure 4), but the difference was significant in both cases (*p* = 0.001 and 0.007, respectively). On average across treatments, Mv Hópehely, Mv Istráng, and GK Arany clearly stood out from the other varieties, with higher N contents (2.51%, 2.50%, and 2.47%, respectively). No significant differences in nitrogen content were measured between the varieties in the plant samples (1.30%–1.47%).

**Figure 5:**
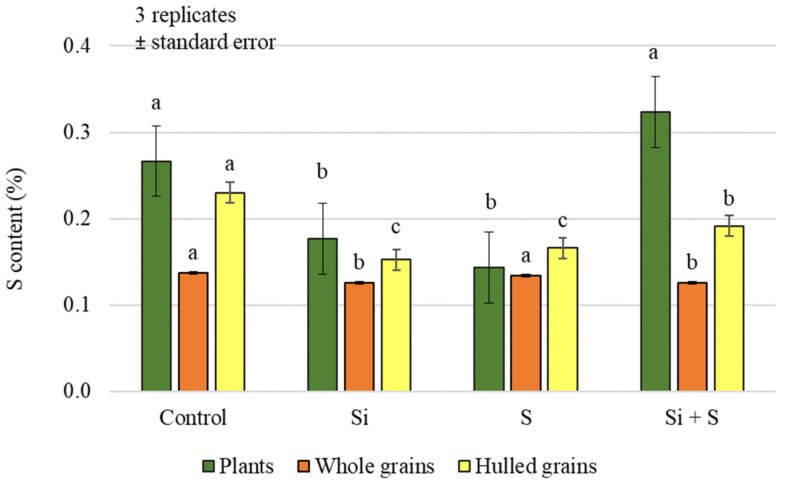
Sulphur content of the winter oat plants, and whole and hulled grains, as a function of foliar fertilisation treatments; ± standard error of means. 3 replicates. The different letters indicate significant differences between the treatments, separately in different plant samples, at *p* = 0.05 level.

**Figure 6:**
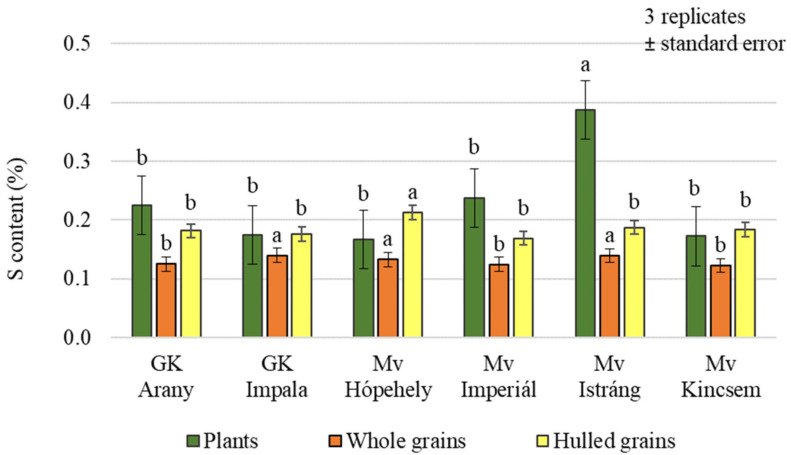
Sulphur content of the winter oat varieties (plants, whole and hulled grains); ± standard error of means. 3 replicates. The different letters indicate significant differences between the varieties, separately in different plant samples, at *p* = 0.05 level.

Foliar treatments had a significant effect on the sulphur (S) content of both plant samples and grains, but no difference could be demonstrated between the control and Si + S, and between S and Si treatments, in pairwise comparisons in plant and hulled grain samples (Figure 5). In whole grain analysis, a significantly higher sulphur content was found in the control and sulphur-treated plots (0.138%, 0.134%, respectively) compared to the Si and Si + S treatments (0.125%, 0.126%, respectively). The difference does not seem large, but the standard deviation was very small (0.000416–0.012201), so the difference can be statistically justified. There was no significant difference in the hulled grain sulphur content of the varieties (*p* = 0.451). The post hoc test could only confirm the difference between the varieties with the lowest (Mv Imperiál; 0.169%) and the highest sulphur contents (Mv Hópehely; 0.213%). In contrast, whole grain’s sulphur content was significantly different between varieties, although in pairwise comparisons, GK Arany did not differ from GK Impala and Mv Kincsem, GK Impala from Mv Istráng, and Mv Imperiál from Mv Kincsem (Figure 6). The foliar fertiliser treatments caused greater differences in both carbon and nitrogen and sulphur contents in the green plant samples, compared to the differences measured in the grain.

### 2.2. Effect of Silicon and Sulphur Treatments on the Protein Content of Oat Whole Grains

**Figure 7:**
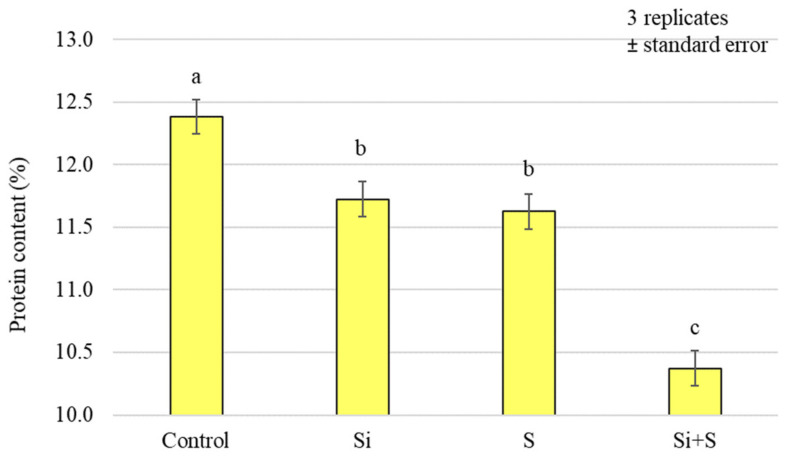
Protein content of the winter oat whole grains as a function of foliar fertilisation treatments; ± standard error of means. 3 replicates. The different letters show significantly differing values, at *p* = 0.05 level.

When we analysed the protein content of the whole grains, it was found that, similar to the nitrogen content, the control plots had the highest value (12.38%), and the (Si + S)-treated plots the lowest (10.37%). The treatments caused a significant difference in protein content (*p* < 0.001), but there was no detectable difference between the Si and S treatments. The standard deviation was quite small, ranging from 0.34% to 5.25% of the mean (Figure 7).

**Figure 8:**
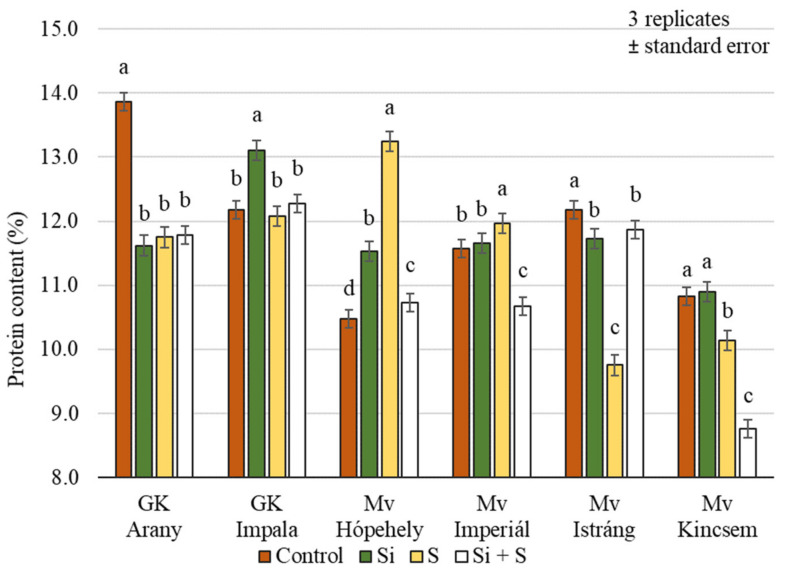
Whole grains protein content of the winter oat varieties as a function of foliar fertilisation treatments; ± standard error of means. 3 replicates. The different letters show significantly differing values among the treatments in the varieties, at *p* = 0.05 level.

Significant differences (*p* = 0.001) in the protein content were identified between winter oat varieties in response to treatments, but the varieties did not respond in the same way to different foliar fertiliser treatments (Figure 8). For GK Arany and Mv Istráng, the highest protein content was measured in the control plot, while for GK Impala and Mv Kincsem the highest protein content was measured in the Si treatment, and for Mv Hópehely and Mv Imperiál in the S treatment. The protein content of Mv Kincsem was low compared to the other varieties (10.41%), while the highest value was measured in the GK Impala variety (12.41%), averaged over the treatments.

### 2.3. Effect of Silicon and Sulphur Treatments on the Element Contents of Oat Whole Grains

Significant differences in the Al, Ba, Ca, Cu, Fe, K, Mn, Mo, Na, Ni, P, Pb, Sr, and Zn contents of oat whole grains were measured both between treatments and between varieties (*p* = 0.001–0.006). No significant differences in Mg (*p* = 0.657), Li (*p* = 0.507), and B (*p* = 0.083) content were found between the differently treated plots (Table 1). Al, Ba, Li, Mo, Na, Ni, and Pb concentrations in the oat grains were highly variable (0.01–2.63 mg kg^−1^; 0.57–1.96 mg kg^−1^; 0.05–0.2 mg kg^−1^; 0.98–2.32 mg kg^−1^; 21.60–95.20 mg kg^−1^; 0.76–2.46 mg kg^−1^; and 0.01–0.84 mg kg^−1^, respectively), while Ca, K, Mg, Mn, P, and S contents varied least (797.33–1071.33 mg kg^−1^; 3630.00–4591.00 mg kg^−1^; 1246.67–1671.00 mg kg^−1^; 49.83–64.56 mg kg^−1^; 2485.33–3479.67 mg kg^−1^; and 1045.33–1552.67 mg kg^−1^, respectively).

Similar to our findings for the nitrogen, sulphur, and protein contents of oat grains, where the control plots had the highest values, the control plots also had the highest values for the Al, B, Ca, Fe, K, Na, P, Pb, S, Sr, and Zn contents. The plots with the sulphur treatment had the highest contents of Ba, Cu, Mg, and Ni in the grains, while those with the Si + S treatment had the highest contents of Li, Mn, and Mo. The Fe:Mn ratio was also influenced by the foliar fertiliser treatments, and differences between the cultivars were also detected. The ratio was highest in the control treatment and decreased in the order of Si, S, Si + S treatments (0.785, 0.736, 0.689, and 0.674, respectively). Among the varieties, the highest Fe:Mn ratio, of 0.934, was found in the grain yield of GK Impala, while the lowest was found in Mv Kincsem, 0.607. The high Na content of the samples was accompanied by a high K and low Mo content. This finding is confirmed by the Pearson correlation coefficient values, as shown in Table 2.

Phosphorus and potassium were found in large concentrations (2485.33–3479.67 and 3630.00–4591.00 mg kg^−1^, respectively) in oat grains, compared to the other elements, which may have been influenced by the exceptionally high phosphorus (AL-soluble P_2_O_5_ 1076.8–1671.6 mg kg^−1^) and potassium (AL-soluble K_2_O 525.5–658.9 mg kg^−1^) contents of the soil.

**Figure 9:**
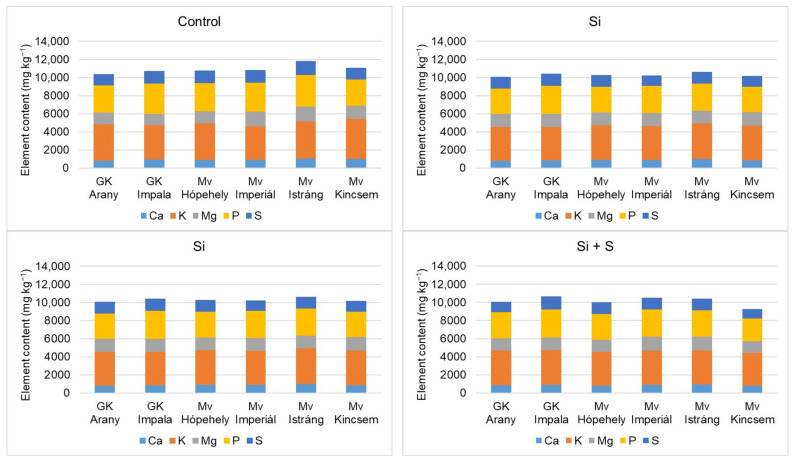
Whole grain Ca, K, Mg, P, and S contents of the winter oat varieties as a function of the foliar fertilisation treatments.

**Figure 10:**
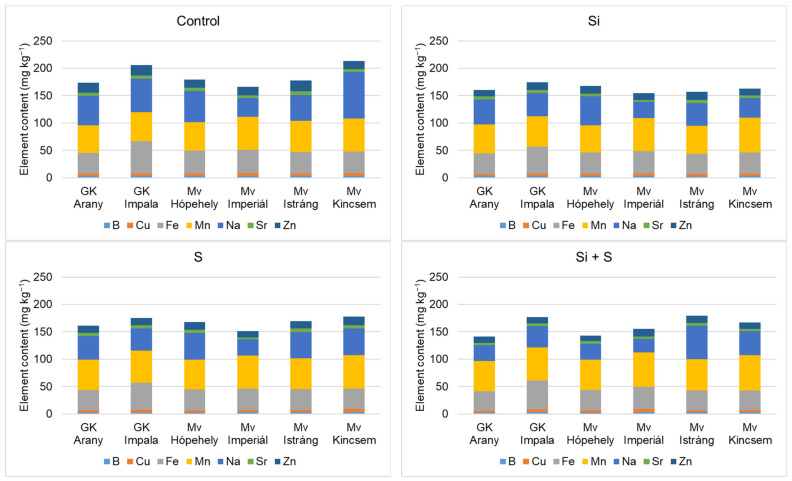
Whole grain B, Cu, Fe, Mn, Na, Sr, and Zn contents of the winter oat varieties as a function of the foliar fertilisation treatments.

Figure 9 and Figure 10 show that the winter oat varieties did not respond equally to the foliar fertiliser treatments in terms of either macronutrient or micronutrient contents. When P, K, Ca, Mg, and S were summarised, the control treatment was the best. Among the varieties, Mv Istráng stood out in the control, Si, and S foliar fertilisation treatment plots, while Mv Kincsem was second. In the Si + S treatment, the GK Impala variety had the highest combined P, K, Ca, Mg, and S contents. Mv Imperiál had the highest Mg content in the grains, and also the highest Mn and Mo concentrations (Figure 6 and Figure 7). The black-seeded winter oat variety Mv Imperiál had average values, not significantly different from the other varieties.

GK Impala had significantly higher Cu and Fe contents compared to the other varieties. The highest zinc content was measured in the grains of Mv Istráng, with the exception of the S-treated plots, where Mv Kincsem had the highest zinc content (16 mg kg^−1^).

Table 2 shows the results of the Pearson correlation analysis. The analysis showed a positive, moderate but significant relationship between Al and P, S, and Zn content (r = 0.428, 0.405, 0.440, respectively), B and Ni content (r = 0.408), Cu and Fe content (r = 0.423), and Ba and Ni content (r = 0.477).

### 2.4. Results of the Discriminant Analysis

The eigenvalues and the structure matrix of the canonical discriminant functions show that function 1 represented 58.5% of the variance (Zn, protein, Mo, Na, P, Al, Ca, Fe, and Li), while function 2 represented 30.1% (Pb and B), and function 3 represented 11.4% (K, S, Ni, Cu, Sr, Mn, Ba, and Mg).

**Figure 11:**
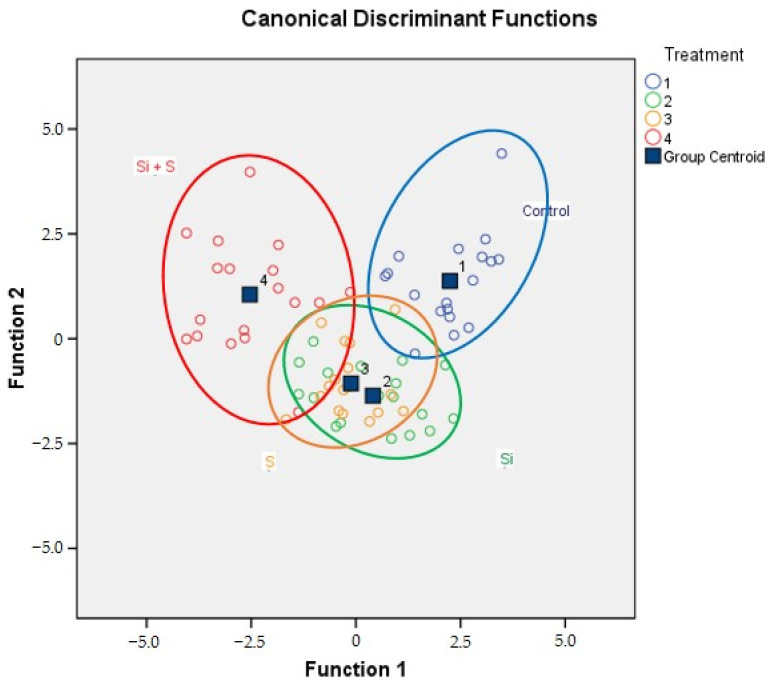
The combined groups plot of discriminant analysis for foliar fertiliser treatments (Debrecen, 2021). (Treatment 1: Control; Treatment 2: Si; Treatment 3: S; Treatment 4: Si + S).

The results of the discriminant analysis on the foliar fertilisation data are shown in Table 3. The classification was successful, 86.1% of the original grouped cases were correctly classified. The share of the correctly classified cases in the case of the control, Si, S, and Si + S treatments were 94.4%, 77.8%, 83.3%, and 88.9%, respectively. On the combined groups plot (Figure 11), the fertilisation treatments can be separated visually, but overlapping can also be observed, especially on the Si and S treatments.

## 3. Discussion

Sustainable winter oat production can rely on a continuous renewal of fertiliser use, especially when the amount of available nutrients limits the growth and development of winter oats under changing environmental conditions. Application of synthetic chemical fertilisers is required to enhance the oat yield and ameliorate grain nutritional profiles, while maintaining the fertility of the soil. Wheat and rice are the most widely consumed cereals, with people consuming much higher amounts than oats [25]. However, oats have the advantage of being generally consumed as a whole grain, and whole grain oats contain significant amounts of valuable nutrients such as proteins, starch, unsaturated fatty acids, and dietary fibre, in the form of soluble and insoluble fractions [26].

Silicon accumulation is known to decrease the carbon content of the aboveground tissues of plants, especially grasses like rice. Liu et al. [27] reported that, in their research, the C content of plants was negatively correlated with the Si content of plants, but carbon accumulation increased due to more Si being stored. Their results also suggest that Si plays an important role in regulating plant C accumulation and long-term C storage in salt-tolerant Si accumulators. We found a similar result in our study, that Si treatment mainly affected the C content of grains rather than green plant samples, and the control plots had the highest values. On the other hand, the sulphur treatment significantly increased the carbon content of the green plants, although no such correlation was found for hulled grains. 

We measured the N content in both green plant parts and grains, similar to the results of previous studies [28]. The genetic diversity of the varieties is shown by the statistically significant differences in grain N and protein contents between the varieties. Our data support the observations of several researchers [29], that the quality parameters of oat grains are essentially determined by the genetic backgrounds of the varieties. This confirms the importance of variety choice.

Oats are a good source of protein, having a distinctive protein makeup and a high protein concentration, of 11–15% [5]. It is well known that protein content and protein composition are strongly influenced by growing conditions. Gell et al. [7] found that for the 180 oat varieties they analysed, the protein content of samples of the same variety could vary by 15 percent with water availability. Studies on Si [30] and S-treatments [31,32] report that protein content generally increases as a result of the treatment. The results of Helal et al. [30] confirm this. Silicon treatment induced an increase in protein content and Ca and K levels in wheat exposed to heat stress. In conclusion, pronounced positive effects, associated with improved yield and quality, were observed in wheat treated with Si particles and exposed to stress. In contrast, we found that the protein content of whole oat grains was highest in the control plots and lowest in the (Si + S)-treated plots, on average across the six varieties. The treatments caused a significant difference in protein content, but there was no detectable difference between the Si and S treatments. In contrast, two varieties (GK Impala and Mv Kincsem) had the highest protein content in plots treated with Si. In accordance with other researchers [33], we also found significant differences in protein content between the investigated winter oat varieties.

Synergistic and antagonistic effects between the applied nutrients and the P and micronutrient content of the grain can be observed due to the application of sulphur and silicon, either alone or in combination. Although phosphorus and micronutrient concentrations varied with the genotype of the varieties studied, our results suggest that silicon, sulphur, and a combination of both, with the exception of Ba, Li, Mn, and Mo, may result in lower element deposition in oat grains. This is in contrast to results reported by other researchers, where Si fertilisation increased the microelement content, with the exception of zinc, of grains in rice [34] (rice is known to be a Si accumulator plant).

In our experiments, foliar fertiliser treatments significantly reduced the Ca, P, and Zn contents of the samples, with lower values compared to the control, for the Si, S, and Si + S treatments. The potassium content was significantly decreased only in plots receiving the Si and Si + S treatments compared to the control, the S treatment did not change the K content. This partially contradicts the results of Helal et al. [30], who observed an increase in the Ca, P, and Zn contents with Si treatment in two wheat cultivars. The Zn content was relatively low (11.921–17.083 mg kg^−1^) compared to the findings of other researchers [35,36,37,38], which was probably caused by the low Zn content (1.84–2.77 mg kg^−1^) in the soil of the experiment area. In contrast, Alemayehu [33], who studied oat landraces and varieties grown in Ethiopia, found Zn levels similar to ours (16–20 mg kg^−1^). The application of sulphur significantly decreased the Fe:Mn ratio in the grains, and this effect was also confirmed when applied together with Si. This is in agreement with the results of Barczak et al. [23].

In plants, manganese acts as an activator for over a hundred enzymes, and plays an important role in lipid and carbohydrate metabolism. The Mn content of cereals (wheat, oats, barley, and rye) is influenced by a number of factors, one of the most important being soil pH. According to the literature, a low soil pH means higher Mn uptake by crops, which in turn increases the Mn content of the grains [39]. The soil in our experiment is not acidic, but we measured 55.561–59.194 mg kg^−1^ Mn in oat grains, which is higher than reported by Bityutskii, (3.5–9.9 mg kg^−1^) [37].

Examining the relationships between phosphorus and protein content in the grain yield of winter oat varieties, we found that the winter oat variety with the lowest P concentration (GK Arany) accumulated the highest grain protein content, even under unfertilised conditions. Thus, understanding the relationship between these two traits may be important for breeding winter oat varieties and improving phosphorus fertilisation strategies.

Si application in deficient conditions can improve nutrient uptake by most plants, for example by increasing nitrogen, potassium, and iron uptake. More importantly, Si appears to play an important role in the biogeochemical cycling of carbon and nutrients in arable fields [40]. Previous studies, and our results, have shown that higher nutrient uptake in Si-treated plants is observed in plants under stress [41]. Walsh et al. [42] reported that there was no significant effect of Si rate and application time on plant height and nutrient uptake of irrigated winter wheat grown under non-stressed conditions.

## 4. Materials and Methods

### 4.1. Soil Characteristics of the Experimental Site

The field experiment was founded by Dr. Erika Tünde Kutasy in the autumn of 2020 in Debrecen, Hungary, in the experimental garden of the University of Debrecen (coordinates of 47°33′02″ N; 21°35′56″ E). The area has Calcic–Endofluvic–Chernozem soil [43], with a good humus content (Hu% = 2.7–3.66), a slightly alkaline (pH_H2O_ = 8.3–8.43) acidity, and a high content of available phosphorus (AL-soluble P_2_O_5_ 1076.8–1671.6 mg kg^−1^) and potassium (AL-soluble K_2_O 525.5–658.9 mg kg^−1^), as shown in Table 4. The soil analysis was made at the Agricultural Laboratory Centre of the University of Debrecen. The laboratory is accredited by the National Accreditation Board of Hungary.

### 4.2. Climatic Conditions 

A detailed analysis of the climatic conditions, and the figures attached as Supplementary Materials, were published in our previous article [1]. In the experimental year, the oat stands were severely affected by drought, which allowed testing of the abiotic stress tolerance of the varieties. In 2021, June was extremely dry, with only 6 mm of precipitation, compared to the 30-year average of 66.5 mm, and an average temperature that was 3.2 °C higher than the 30-year average (Figure S1). Even in March, the AET/PET ratio was low (55–56%). The ratio ranged between 41 and 46% in April and May. In June, long drought periods were recorded without precipitation (AET/PET ratio 30–44%) (Figure S2). These low values demonstrate that the very low available moisture content in the soil limited the evapotranspiration. At 10 cm depth, the soil was drier (9.7–15 V%) than the permanent wilting point (15.2 V%). The water deficit varied between 110.4–122.9 mm in the 0–60 cm layer in June (Figure S3).

### 4.3. Experimental Setup

The winter oat small plot (1.5 × 7 m = 10.5 m^2^) experiment, with three independent repetitions, was sown on 26 October 2020, applying 550 seeds per m^2^ seed rate, with the depth of 5 cm. Fertilisation was: N_20_P_40_K_120_ kg ha^−1^, October 2020, N_50_ kg ha^−1^, 26 February 2021. The forecrop was potato, harvested in September 2020. In the experiment the applied chemical protections were: 

Seed dressing: tebukonazol

Weed control: tribenuron-metil + tifenszulfuron-metil. 19 April 2021, at end of tillering (BBCH29 according to Meier [44])

Pest control: alfametrin 0.1 L ha^−1^ against cereal leaf beetle (*Oulema melanopus*). 19 April 2021, at end of tillering (BBCH29), and 3 June 2021, at end of flowering (BBCH69)

We applied 4 foliar fertiliser treatments:

1. Control, without foliar fertilisation
2. Silicon fertilisation (Si) 3.0 L ha^−1^
3. Sulphur fertilisation (S) 5.0 L ha^−1^
4. Silicon + sulphur fertilisation (Si + S) 3.0 + 5.0 L ha^−1^

Foliar fertilisers:

- Sulphur fertiliser: liquid foliar fertiliser with high content sulphur (lignosulfonate formulation) 1000 g L^−1^ SO_3_, 30 g L^−1^ N, 30 g L^−1^ MgO, 27 g L^−1^ B, 0.003 g L^−1^ Mo
- Silicon fertiliser: (potassium silicate formulation) 1.4 m/m% Si, 10.5 m/m% K_2_O

Foliar fertilisation application times:

1 December 2020BBCH13 (3 leaves unfolded)10 May 2021BBCH39 (flag leaf stage)18 June 2021BBCH77 (late milk)

The six tested winter oat (*Avena sativa* L.) genotypes were, ‘Mv Hópehely’, ‘Mv Kincsem’, ‘Mv Imperiál’, ‘Mv Istráng’, ‘GK Arany’, and ‘GK Impala’.

The grain yield of each plot was harvested by Wintersteiger 125 plot combined with 125 cm cutting width. We took grain samples from each plot to determine the grain moisture content and 1000 kernel weight (published in our previous article [1]), grain protein content and element content.

### 4.4. Measurements, Calculations, and Their Methodology

#### 4.4.1. Plant Carbon, Nitrogen, and Sulphur Contents

Plant samples were collected on 24 June (BBCH 77). Total carbon, nitrogen, and sulphur (CNS) concentrations of the plant samples were determined by a dry combustion method [45,46]. A Vario Macro Cube CNS analyzer (Elementar Analysensysteme GmbH, Hanau, Germany) was used for plant analysis. Before analysis, plant samples were dried, homogenised, and finely ground, then samples were weighed into specific foil tins. Tungsten trioxide powder was added to the sample in a ratio of 1:1 to bind the earth alkaline/alkaline ions. Sulfanilamide, as a standard material, was used to determine the daily measurement factor of the equipment (calibration). Samples from the three repetitions were homogenised during preparations, hence one measurement represents a certain genotype under a certain treatment in all repetitions.

#### 4.4.2. Grain Carbon, Nitrogen and Sulphur Content

Analogously to plant CNS content determination, a Vario Macro Cube CNS analyzer (Elementar Analysensysteme GmbH, Hanau, Germany) was used, and the method is based on dry combustion methods [45,46]. In the first phase, grains were manually separated from the husk using a sharp knife and tweezers, and were dried out [47,48,49,50]. For each sample we prepared 100 grains this way, which were then finely ground, applying an electric grinder, and the resultant powder was weighed into specific foil tins. Tungsten trioxide powder was added to the sample in a ratio of 1:1 to bind the earth alkaline/alkaline ions. Sulfanilamide, as a standard material, was used to determine the daily measurement factor of the equipment (calibration). Samples from the three repetitions were homogenised during preparations, hence one measurement represents a certain genotype under a certain treatment in all repetitions.

#### 4.4.3. Grain Protein Content by Kjeldahl Method

The Kjeldahl method was used to determine the nitrogen content of the oat grain samples [51]. We measured 1 g from the sample on the nitrogen-free paper (with 4 tenth accuracy- 0.0000), and put it into the digestion tube, adding two catalyst tablets (Kjeltabs S/3,5) and 14 mL sulfuric acid (H_2_SO_4_) to it, and put it into a 420–430 °C block heater to decompose the grain sample by oxidation. Digestion lasted for 2 h, then the samples were allowed to cool. We used a specific (Jones) conversion factor for the conversion of nitrogen content to protein content, which, in the case of oats, is 5.83 [27,52]. 

#### 4.4.4. Grain Element Content

The concentrations of the elements Al, B, Ba, Ca, Cu, Fe, K, Li, Mg, Mn, Mo, Na, Ni, P, Pb, S, Sr, and Zn were determined by using a PerkinElmer Optima 3300 DV Inductively Coupled Plasma Optical Emission Spectrometer (ICP–OES) (PerkinElmer Inc., Waltham, MA, USA). This device is an echelle-grating-based ICP instrument, capable of determining the concentrations of multiple elements simultaneously.

The grain samples were ground, and then they were prepared by wet acid digestion. The digestion included two stages: predigestion and digestion. An amount of 1 g of oat grains was measured into a digestion tube, and 10 mL nitric acid (HNO_3_, 69% *v*/*v*) (VWR International Ltd., Radnor, PA, USA) was added to the sample. After 12 h digestion, the samples were heated to 60 °C for 30 min in a LABOR MIM OE-718/A block digestion apparatus. Following the predigestion, after cooling for a few minutes, 3 cm^3^ of hydrogen-peroxide (H_2_O_2_, 30% *v*/*v*) (VWR International Ltd., Radnor, USA) was added to the samples. In the main digestion phase, the temperature was raised to 120 °C for 90 min. After digestion and cooling of the samples, the volume of the digested liquid was increased to 50 cm^3^ using ultrapore water (Milli Q two-level water purification system, Millipore S.A.S, Molsheim, France), and then filtered using Filtrak 388 filter paper, and then analysed by ICP-OES [53].

### 4.5. Data Analysis

The IBM SPSS Statistics 26.0 statistical software programme (IBM Corp., Chicago, IL, USA) was used for data analysis and evaluation. To compare the means of the different parameters among the varieties and treatments, the univariate GLM model with descriptive statistics turned on was utilised. We tested the pre-requisites for analysis of variance (normality, homogeneous variances, and independency) on the dependent variables. The Kolmogorov–Smirnov test was used to check for normality. For pairwise comparisons of the means, LSD post hoc testing was used. In the statistical analysis, *p* = 0.05 was used as the significance threshold (alpha). To test group membership, discriminant analysis was applied to standardised values. The linear correlations between the parameters were discovered using Pearson correlation analysis (2-tailed).

## 5. Conclusions

The research results reported in this article, an evaluation of the chemical composition of different oat varieties, complement the studies on the quality properties of several oat varieties from around the world, based on six Hungarian varieties.

The treatments used in the experiment had effects on the plant and grain quality parameters of winter oats. Significant differences were measured in the mineral compositions of the tested varieties. The investigated winter oat cultivars did not respond equally to the foliar fertilisation treatments, in terms of both macro- and micronutrient contents.

Application of silicon, sulphur, and the combination of both could result in lower phosphorus deposition in oat grains. A greater difference in phosphorus content was measured between varieties than as a result of the treatments. The observed differences between the applied nutrients, grain nutritional concentration, and divergence in genetic response could be an important target for agronomists and breeders.

Our hypothesis was partially confirmed, as for some elements (Mn, Mo, Li, and Ba) silicon, sulphur, and the combined treatment increased accumulation in the grain, but for most elements the deposition was lower, or did not change significantly (B and Mg) in the treated plots, both in whole or hulled grain and in green plant samples. Protein content was also highest in the untreated plots, although the response of the varieties was very different.

Among the tested varieties, the highest yields in 2021, in both the control and treated plots, were obtained by the varieties GK Arany, Mv Istráng, and Mv Hópehely. These results were published in our previous article [1]. It is noteworthy that the grain nitrogen content of these varieties was also remarkably good. GK Arany had the highest protein content, of nearly 14%, in the untreated plots, but Mv Hópehely was also above 13%, although only in the sulphur-treated plots. Of course, we cannot draw any firm conclusions based on one year’s experimental results, but we continued our experiments in 2022. The highest yields were also achieved by the same three varieties in this year, but as 2022 was even drier and more unfavourable, the yield levels were slightly lower [54]. In 2021, all three treatments significantly increased yields, but in 2022 only the combined silicon and sulphur treatment showed a significant increase. We have not yet had the opportunity to evaluate the 2022 quality results, which will be published later. We will continue the experiment in 2023, which will allow further correlations to be explored.

Based on our results, we can influence the plant and grain quality parameters of winter oats with foliar fertiliser treatments. On this basis, we recommend to oat growers the use of the foliar fertilisers in drought-prone areas, taking into account that we measured significant differences in the responses and mineral compositions of the varieties.

## Figures and Tables

**Table 1:** The element content (mg kg^−1^) of winter oat whole grains under the influence of different foliar fertilisation treatments, mean of varieties, ± standard error (Debrecen, 2021).

	Control	Si	S	Si + S
Al	1.396 ± 0.162 a	1.017 ± 0.188 b	0.972 ± 0.130 b	0.671 ± 0.132 c
B	3.070 ± 0.071 a	2.833 ± 0.085 a	2.783 ± 0.117 a	2.882 ± 0.129 a
Ba	0.895 ± 0.057 c	1.007 ± 0.081 b	1.121 ± 0.114 a	1.072 ± 0.097 b
Ca	943.00 ± 23.73 a	894.00 ± 18.85 b	903.83 ± 18.81 b	854.67 ± 16.94 c
Cu	4.373 ± 0.078 a	4.082 ± 0.095 b	4.394 ± 0.149 a	4.048 ± 0.193 b
Fe	43.456 ± 1.889 a	40.689 ± 1.312 b	39.778 ± 1.041 b	39.822 ± 1.527 b
K	4002.61 ± 69.15 a	3792.06 ± 27.46 b	3947.33 ± 59.20 a	3779.06 ± 26.87 b
Li	0.134 ± 0.004 b	0.136 ± 0.005 b	0.138 ± 0.008 b	0.142 ± 0.009 a
Mg	1444.89 ± 44.20 a	1422.56 ± 28.27 a	1453.94 ± 25.33 a	1399.83 ± 30.99 a
Mn	55.794 ± 1.017 c	55.561 ± 1.320 c	57.750 ± 1.118 b	59.194 ± 1.153 a
Mo	1.416 ± 0.059 c	1.534 ± 0.052 c	1.572 ± 0.086 b	1.869 ± 0.081 a
Na	56.461 ± 4.012 a	41.167 ± 2.205 b	43.250 ± 1.955 b	37.706 ± 3.243 c
Ni	1.492 ± 0.075 a	1.306 ± 0.073 c	1.527 ± 0.069 a	1.395 ± 0.100 b
P	3168.94 ± 50.40 a	2926.89 ± 55.05 c	3015.50 ± 40.57 b	2869.72 ± 53.22 c
Pb	0.377 ± 0.057 a	0.169 ± 0.042 c	0.134 ± 0.039 c	0.230 ± 0.050 b
S	1375.39 ± 28.40 a	1258.44 ± 22.29 b	1342.00 ± 27.40 a	1254.33 ± 32.21 b
Sr	5.600 ± 0.186 a	5.226 ± 0.193 a	5.535 ± 0.227 a	4.727 ± 0.110 b
Zn	17.083 ± 0.565 a	12.913 ± 0.426 b	13.456 ± 0.404 b	11.921 ± 0.394 c

Different letters mean statistically different values in the rows, at *p* = 5%.

**Table 2:** Pearson correlation coefficient values between element content in winter oat whole grains (Debrecen, 2021).

	Ca	Cu	Mo	Fe	K	Mg	P	S	Zn	Ni	Na
Ca	1	0.213	−0.409 **	0.100	0.528 **	0.235 *	0.646 **	0.539 **	0.540 **	0.075	0.320 **
Cu	0.213	1	−0.007	0.423 **	0.242 *	0.109	0.325 **	0.342 **	0.317 **	0.706 **	0.132
Mo	−0.409 **	−0.007	1	0.169	−0.455 **	0.251	−0.177	−0.159	−0.465 **	0.242	−0.655 **
Fe	0.100	0.423 **	0.169	1	−0.227	−0.018	0.476 **	0.398 **	0.287 *	0.360 **	0.095
K	0.528 **	0.242 *	−0.455 **	−0.227	1	0.070	0.317 **	0.331 **	0.405 **	0.127	0.506 **
Mg	0.235 *	0.109	0.251	−0.018	0.070	1	0.351 **	0.369 **	0.228	0.224	−0.156
P	0.646 **	0.325 **	−0.177	0.476 **	0.317 **	0.351 **	1	0.774 **	0.683 **	0.195	0.143
S	0.539 **	0.432 **	−0.159	0.398 **	0.331 **	0.369 **	0.774 **	1	0.575 **	0.114	0.208
Zn	0.540 **	0.317 **	−0.465 **	0.287 *	0.405 **	0.228	0.683 **	0.575 **	1	0.126	0.420 **
Ni	0.075	0.706 **	0.242	0.360 **	0.127	0.224	0.195	0.114	0.126	1	−0.019
Na	0.320 **	0.132	−0.655 **	0.095	0.506 **	−0.156	0.143	0.208	0.420 **	−0.019	1

*: the correlation is significant at *p* = 5%; **: the correlation is significant at *p* = 1%.

**Table 3:** Discriminant analysis classification results of fertilisation treatments.

Treatment	Predicted Group Membership			
	Control	Si	S	Si + S
Control	94.4%	0%	5.6%	0%
Si	0%	77.8%	22.2%	0%
S	0%	11.1%	83.3%	5.6%
Si + S	0%	11.1%	0%	88.9%

86.1% of original grouped cases correctly classified.

**Table 4:** Soil analysis results of the experiment area (2021, Debrecen), first published by Kutasy et al. [1].

	Layer 0–20 cm	Layer 20–40 cm	Layer 40–60 cm
pH (H_2_O)	8.30	8.36	8.43
K_A_	38	38	38
CaCO_3_ (%)	8.1	8.1	8.1
Humus (%)	3.66	2.92	2.70
NO_3_ + NO_2_ (mg kg^−1^)	1.71	2.95	3.18
NH_4_ (mg kg^−1^)	0.84	1.02	3.18
P_2_O_5_ (AL) (mg kg^−1^)	1671.6	1376.1	1076.8
K_2_O (AL) (mg kg^−1^)	658.9	648.2	525.5
SO_4_ (mg kg^−1^)	3.07	6.00	7.81
Zn (EDTA) (mg kg^−1^)	2.77	2.24	1.84

Note: K_A_: Arany-type plasticity; AL: ammonium lactate-soluble; EDTA: ethylenediamine tetraacetic acid-extractable.

## Data Availability

The data presented in this study are available on request from the corresponding author.

## Supplementary Materials

The following supporting information can be downloaded at: https://www.mdpi.com/article/10.3390/plants12040969/s1, Figure S1: Monthly sums of precipitation and averages of temperature compared to the 30 years’ average (Debrecen, 2020/2021). Figure S2: Potential (PET) and actual (AET) evapotranspiration, AET/PET ratio (%) and VPD (Vapour Pressure Deficit) in oats from March to June (Debrecen, 2021). Average of the decades. Figure S3: Soil moisture content in the 0–100 cm layer in the oat experiment from 3 to 24 June. (Debrecen, 2021).
