# Supplementary material for: Changes in Plant and Grain Quality of Winter Oat (Avena sativa L.) Varieties in Response to Silicon and Sulphur Foliar Fertilisation under Abiotic Stress Conditions
Source: Plants (Basel). 2023 Feb 20;12(4):969. doi: 10.3390/plants12040969 (PMC9967263; doi:10.3390/plants12040969)
Supplement: Supplementary file 1 [file plants-12-00969-s001.zip › Supplementary material_2.pdf]

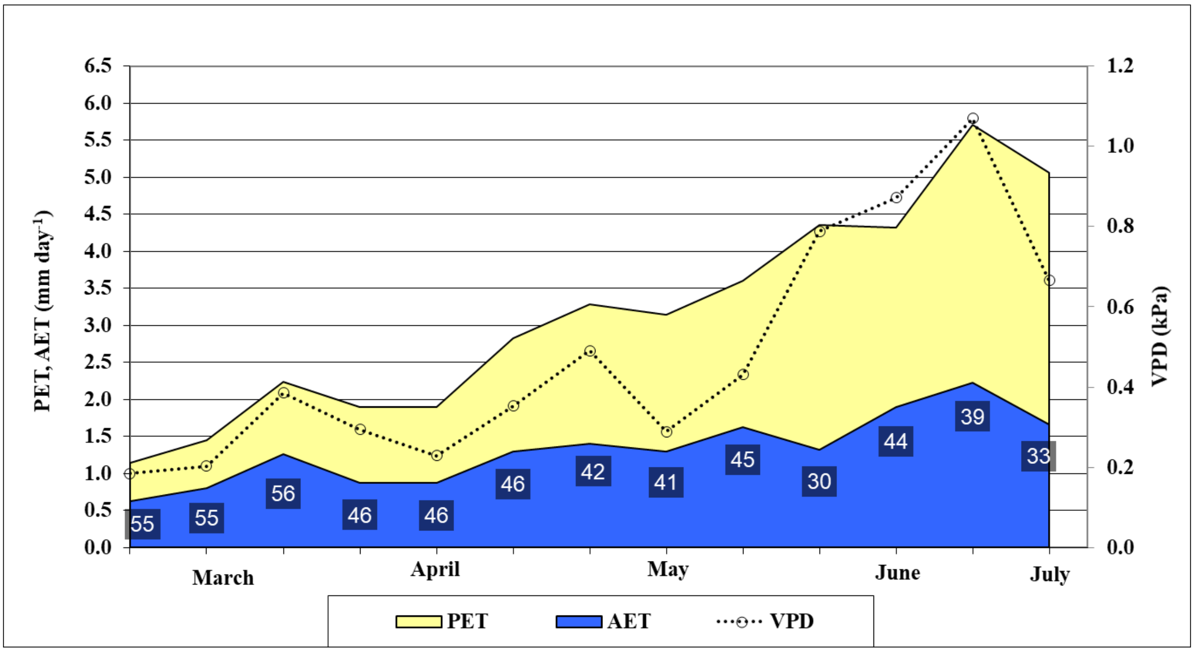

Figure S2. Potential (PET) and actual (AET) evapotranspiration, AET/PET ratio (%) and VPD in oats from March to June (Debrecen, 2021). Average of the decades. VPD: Vapour pressure deficit, calculated using meteorological data.
