# Supplementary material for: Changes in Plant and Grain Quality of Winter Oat (Avena sativa L.) Varieties in Response to Silicon and Sulphur Foliar Fertilisation under Abiotic Stress Conditions
Source: Plants (Basel). 2023 Feb 20;12(4):969. doi: 10.3390/plants12040969 (PMC9967263; doi:10.3390/plants12040969)
Supplement: Supplementary file 1 [file plants-12-00969-s001.zip › Supplementary material_1.pdf]

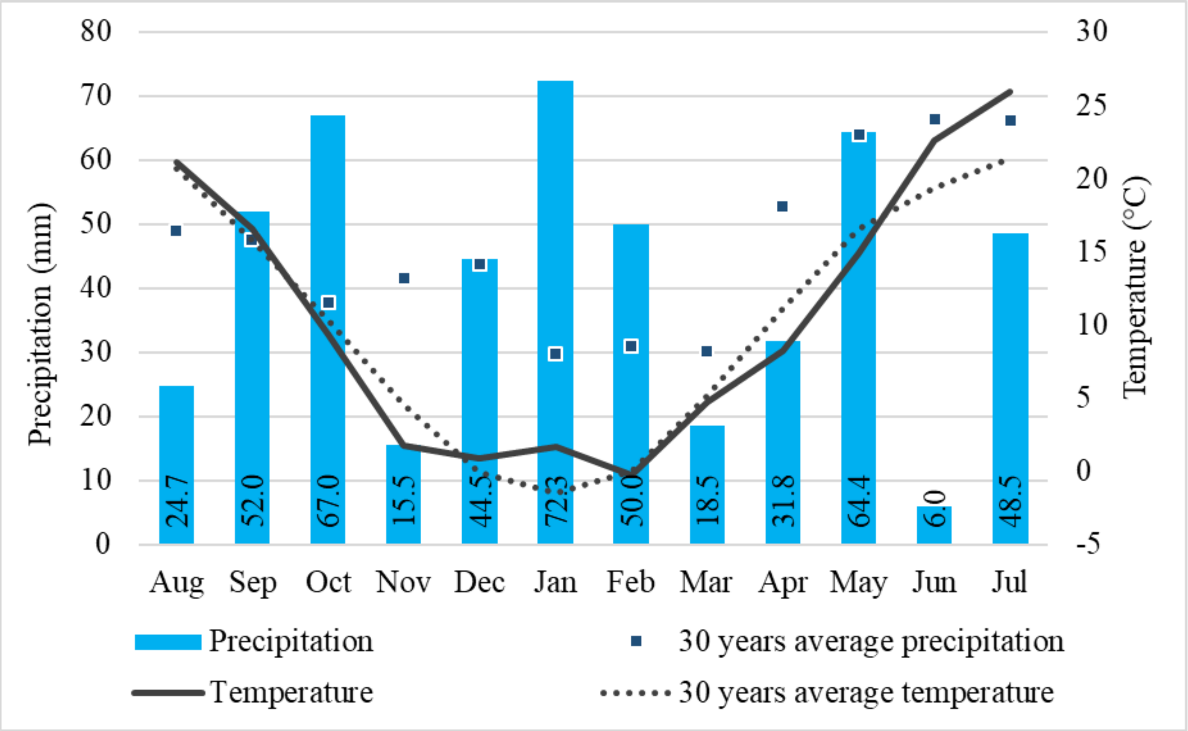

Figure S1. Monthly sums of precipitation and averages of temperature compared to the 30 years' average (Debrecen, 2020/2021). Numbers in the bars: sum precipitation of the month. 30 years' average: monthly averages of the period 1981–2010.
