# Supplementary material for: Changes in Plant and Grain Quality of Winter Oat (Avena sativa L.) Varieties in Response to Silicon and Sulphur Foliar Fertilisation under Abiotic Stress Conditions
Source: Plants (Basel). 2023 Feb 20;12(4):969. doi: 10.3390/plants12040969 (PMC9967263; doi:10.3390/plants12040969)
Supplement: Supplementary file 1 [file plants-12-00969-s001.zip › Supplementary material_3.pdf]

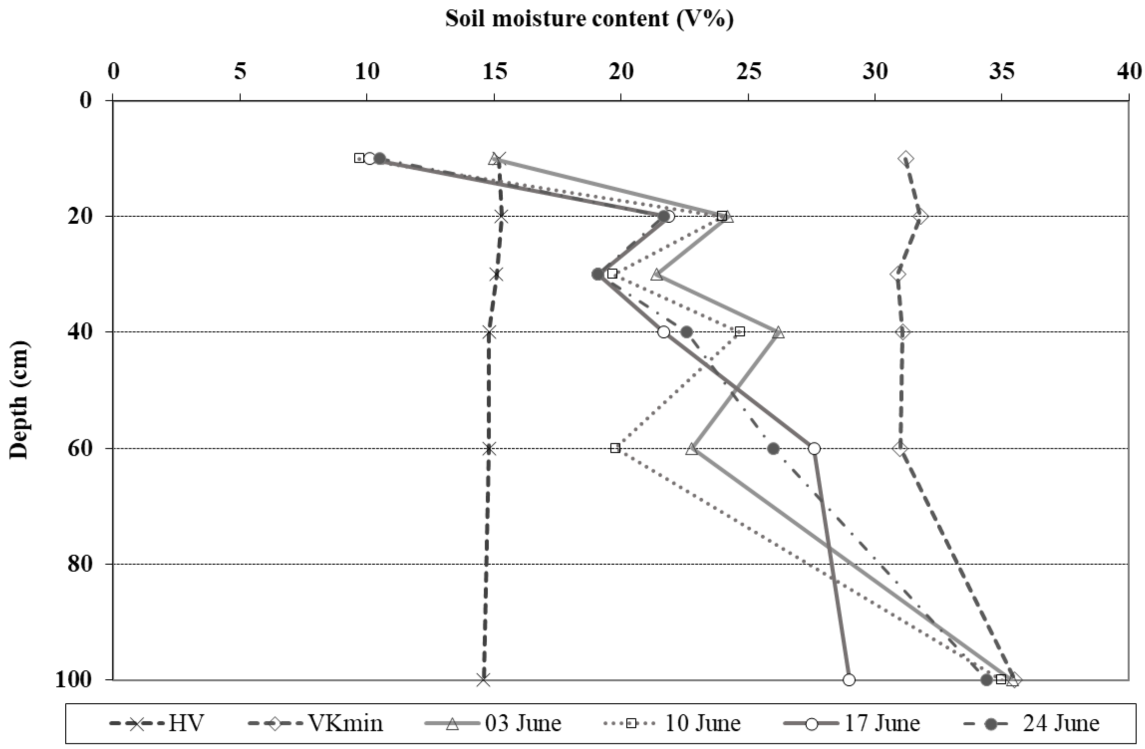

Figure S3. Soil moisture content in the 0 – 100 cm layer in the oat experiment from 3 to 24 June. (Debrecen, 2021). WP: permanent wilting point; FC: field capacity .
